# Development and Content Validation of a Questionnaire for Measuring Beliefs About Using Nicotine Replacement Therapy for Smoking Cessation in Pregnancy

**DOI:** 10.1093/ntr/ntad030

**Published:** 2023-03-02

**Authors:** Joanne Emery, Lisa McDaid, Tim Coleman, Sue Cooper, Ross Thomson, Darren Kinahan-Goodwin, Anne Dickinson, Lucy Phillips, Miranda Clark, Katharine Bowker, Emma Brown, Felix Naughton

**Affiliations:** School of Health Sciences, University of East Anglia, Norwich, UK; School of Health Sciences, University of East Anglia, Norwich, UK; Division of Primary Care, University of Nottingham, Nottingham, UK; Division of Primary Care, University of Nottingham, Nottingham, UK; Division of Primary Care, University of Nottingham, Nottingham, UK; Division of Primary Care, University of Nottingham, Nottingham, UK; Adult Social Care and Health, Derbyshire County Council, Matlock, UK; Division of Primary Care, University of Nottingham, Nottingham, UK; Division of Primary Care, University of Nottingham, Nottingham, UK; Division of Primary Care, University of Nottingham, Nottingham, UK; Division of Primary Care, University of Nottingham, Nottingham, UK; School of Social Sciences, Leeds Beckett University, Leeds, UK; School of Health Sciences, University of East Anglia, Norwich, UK

## Abstract

**Introduction:**

Improving adherence to nicotine replacement therapy (NRT) in pregnancy may result in higher smoking cessation rates. Informed by the Necessities and Concerns Framework, we developed an intervention targeting pregnancy NRT adherence. To evaluate this, we derived the NRT in pregnancy necessities and concerns questionnaire (NiP-NCQ), which measures perceived need for NRT and concerns about potential consequences.

**Aims and Methods:**

Here we describe the development and content validation of NiP-NCQ. From qualitative work, we identified potentially modifiable determinants of pregnancy NRT adherence and classed these as necessity beliefs or concerns. We translated these into draft self-report items and piloted items on 39 pregnant women offered NRT and a prototype NRT adherence intervention, assessing distributions and sensitivity to change. After removing poorly performing items, smoking cessation experts (*N* = 16) completed an online discriminant content validation (DCV) task to determine whether retained items measure a necessity belief, concern, both, or neither construct.

**Results:**

Draft NRT concern items encompassed safety for the baby, side effects, too much or insufficient nicotine, and addictiveness. Draft necessity belief items included perceived need for NRT for short- and longer-term abstinence, and desire to minimize or cope without NRT. Of 22 out of 29 items retained after piloting, four were removed following the DCV task: three were judged to measure neither construct and one possibly both. The final NiP-NCQ comprised nine items per construct (18 total).

**Conclusions:**

The NiP-NCQ measures potentially modifiable determinants of pregnancy NRT adherence within two distinct constructs and may have research and clinical utility for evaluating interventions targeting these.

**Implications:**

Poor adherence to NRT in pregnancy may result from low perceived need and concerns about consequences; interventions challenging these beliefs may yield higher smoking cessation rates. To evaluate an NRT adherence intervention informed by the Necessities and Concerns Framework, we developed the NiP-NCQ. Through the content development and refinement processes described in this paper, we derived an evidence-based, 18-item questionnaire measuring two distinct constructs within two nine-item subscales. Higher concerns and lower necessity beliefs indicate more negative NRT beliefs; NiP-NCQ may have research and clinical utility for interventions targeting these.

## Introduction

Smoking during pregnancy is an international public health problem. Prevalence is 13%–25% in high-income countries,^1–5^ where it is a leading avoidable cause of pre- and perinatal adverse events such as miscarriage, stillbirth, prematurity, low birth weight, perinatal, neonatal, and sudden infant death.^6^ In some countries, such as the UK, nicotine replacement therapy (NRT) is widely prescribed for smoking cessation during pregnancy. However, NRT appears to be less effective for smoking cessation in pregnancy than among the general population.^7,8^ In addition to an acceleration in nicotine metabolism in pregnancy,^9,10^ meaning that higher NRT doses may be required for therapeutic benefit, adherence to NRT is notably poor among pregnant women, with evidence from both trials and routine clinical practice showing that only a minority use it for a sufficient duration.^7,11^

Poor medication adherence can be unintentional, such as forgetting doses, unawareness of the correct dosage, or difficulties in accessing services. However, qualitative evidence from pregnant women and their stop-smoking practitioners suggests that intentional nonadherence, underpinned by negative beliefs about NRT, is a major reason for its underuse among this group.^12,13^ The “Necessities and Concerns” Framework^14^ predicts that medication adherence is principally a function of perceived personal need for a treatment (“necessity beliefs”) weighed up against concerns about potential adverse consequences of using it. Based on this framework, the Beliefs about Medicines Questionnaire (BMQ)^15^ was developed to assess medication-specific concerns and necessity beliefs. The BMQ has since been adapted to medicines for a range of long-term health conditions and has established predictive validity.^14,16,17^ BMQ medication-specific concerns and necessity beliefs form separate scales of 5 items each, with scores ranging from 5 to 25 per scale. Higher scores indicate stronger beliefs in each construct, that is, higher concerns and higher necessity beliefs.

We developed an intervention to support NRT adherence in pregnancy, informed primarily by the Necessities and Concerns Framework. As part of this, we wanted to develop and validate an evidence-based NRT Necessities and Concerns Questionnaire, informed by the BMQ, for use as an outcome measure in a trial of the intervention among pregnant women who smoke (ISRCTN16830506).^18^ A novel measure of NRT beliefs in pregnancy was needed for this context as none existed previously. The Wisconsin Beliefs Assessment on Smoking and Cessation (WI-BASC)^19,20^ measures beliefs about cessation medications among nonpregnant smokers, and has some predictive validity evidence,^21^ but pregnant women have specific concerns and necessity beliefs relating to nicotine and nicotine replacement.^12,13^ This paper aims to describe the development and content validation of an NRT in pregnancy necessities and concerns questionnaire (NiP-NCQ). Specific objectives are to: (1) develop draft NRT concern and necessity belief items informed by qualitative evidence, (2) pilot draft items with pregnant women undergoing a prototype NRT adherence intervention, and (3) establish the discriminant content validity of items retained after piloting, removing poorly performing items to create a final scale.

## Methods

### Phase 1: Content Development

#### Identifying Barriers and Facilitators of NRT Adherence in Pregnancy.

In Phase 1, as part of broader intervention development, we undertook new qualitative studies to identify potentially modifiable determinants of NRT adherence in pregnancy in which (1) 20 women were interviewed individually about their previous experiences of using NRT in pregnancy,^22,23^ (2) 19 specialist pregnancy stop smoking practitioners were interviewed in groups about their experiences of supporting NRT use in pregnancy,^23–25^ and (3) an expert group meeting of seven stop smoking service leads and policymakers was held to discuss the issues raised and how NRT support could be improved. Interview and focus group guides were informed by a systematic review investigating pregnant women’s and health professionals’ views on the barriers and facilitators of pregnancy NRT use.^13^

#### Design of Draft Questionnaire Items.

Barriers and facilitators of pregnancy NRT adherence identified in the research above were rated for importance by the research team based on their strength of evidence and likely potential for modification via a behavioral intervention. Eight researchers with expertise in smoking cessation in pregnancy comprised the research team; three agreed on the initial ratings face to face and all agreed on the final ratings in an online meeting, with discrepancies resolved by group discussion. Barriers and facilitators that we classed as high to medium importance, and as intentional and perceptual in nature (conscious beliefs and cognitions) rather than unintentional or practical (eg, skills, resources),^26^ were further classed as necessity beliefs or concerns, where possible, and translated into draft questionnaire items. Draft items were written and revised by the same research team as above. To match the format of the BMQ, items consisted of statements with a 5-point Likert response scale from “1” (“strongly disagree”) to “5” (“strongly agree”), with “3” representing “neither agree nor disagree.” As usual in questionnaire construction, we drafted an excess of initial items with a view to later item reduction. To prevent agreement (“acquiescence”) bias,^27^ some items of each type were intended to be reverse scored, so that some statements represented a lack of concern about NRT or a lack of perceived need for NRT. BMQ item phrasing was adapted where possible, but we found that few BMQ items were directly adaptable to NRT.

#### Patient and Public Involvement Feedback.

We invited three Patient and Public Involvement representatives, from an established panel with lived experience of smoking in pregnancy, to give feedback on draft item clarity (for example, ease of understanding, ambiguity).

### Phase 2: Pilot Testing of Draft Items With Pregnant Women Who Smoke

As part of an intervention optimization study, the methods of which are detailed elsewhere,^28^ item piloting was carried out with pregnant women who agreed to undergo a smoking quit attempt with NRT and to receive a prototype adherence intervention called “Baby, Me & NRT.” Ethical permission was granted by Nottingham 1 Research Ethics Committee (reference 19/EM/0193). Underpinned by the Necessities and Concerns Framework,^14^ and also the Perceptions and Practicalities Approach,^26^ Theoretical Domains Framework^29^ and Behavior Change Wheel,^30^ “Baby, Me & NRT” is a blended (in-person and digital) behavioral intervention designed to effect positive changes in the barriers and facilitators of pregnancy NRT adherence identified through research described in Phase 1.^13,22–25^ Women completed the questionnaire at baseline, prior to receiving NRT and the adherence intervention, and again at the end of the intervention, 28 days after their agreed quit date, to assess the items’ sensitivity to change. Concern and necessity belief items were presented in the same randomly interspersed order for all participants. Questionnaires were completed on paper at the start of study appointments or, during coronavirus disease 2019, online via a link sent by email and SMS. Participants were able to omit items or leave a comment beside any they found difficult to understand.

### Phase 3: Discriminant Content Validation Task

For questionnaires based on theoretical constructs, it is important to establish that items assess their intended construct and are not contaminated by other constructs within or outside of the scale. To further refine and validate the questionnaire, therefore, a discriminant content validation (DCV) task was carried out on items retained from Phase 2. We closely followed the procedure described in Johnston et al.,^31^ in which experts are asked to judge whether items assess their intended construct and others. We aimed for a minimum sample size of 15, calculated by Johnston et al. as appropriate to detect a large effect size (*α* = 0.05, power = 0.8, two-tailed). Ethical permission was granted by the University of East Anglia Faculty of Medicine and Health Sciences Research Ethics Committee (reference 2020/21-097). Potential participants were stopp smoking experts working in UK universities, at postdoctoral level or above, who were known to the research team. They were invited individually by email using a standard invitation with an embedded link to the full participant information, online consent form, and research task.

Using Qualtrics survey software (https://www.qualtrics.com), all retained Phase 2 questionnaire items, plus two previously considered and rejected items from Phases 1 and 2, were presented in a random order to the above experts. The two previously rejected (dummy) items were considered by the research team to measure neither NRT concerns nor necessity beliefs, and were included for comparison with the questionnaire items. Definitions for “concerns” and “necessity beliefs” were presented to participants alongside each item (Supplementary File). Participants were reminded that items might have reversed scoring, so that a statement could represent a concern or lack of concern about NRT, a perceived need or lack of perceived need for NRT. Following the Johnston et al. procedure,^31^ participants were asked to judge whether items measured each of our two theoretical constructs (“yes” or "no"), and to rate their confidence in each judgment (0–100). We included an optional free text box for alternative construct proposals. Participants completed the task at their own convenience and could stop and return to it later if they wished. From piloting on colleagues, task time was estimated to be 15–20 minutes.

### Statistical Analyses

#### Pilot Testing of Draft Items With Pregnant Women Who Smoke.

Summary statistics and plots were collated on individual item distributions at baseline. Given likely non-normality of data, paired Wilcoxon tests (two-tailed) were used to identify before–after intervention differences in item scores (sensitivity to change). Lower-performing items, such as those with extreme baseline scores, a high proportion of “neither agree nor disagree” responses (which can indicate uncertainty), or no sensitivity to change, were considered for removal or revision by the research team.

#### DCV Task.

A confidence rating was yielded per judge, item, and construct. The initial “yes” or “no” judgment determined the valence of each rating, positive or negative (100 expresses maximum confidence that the item assesses the construct and—100 that it does not). Given likely non-normality of data, one-sample Wilcoxon tests (two-tailed) were used to determine whether the average confidence rating for each item across judges was significantly different from 0 for either construct. A significant, positive confidence rating indicates that an item measures that construct, whereas a significant negative or nonsignificant rating indicates that it does not. Items were ranked in descending order of magnitude of their test statistic; those significant and positive only for their intended construct were classed as having good discriminant validity. Items significant on more than one construct, or neither, were classed as lacking in discriminant validity and considered for removal.

## Results

### Phase 1: Content Development

Based on our qualitative research findings, we devised 15 NRT “concern” and 14 NRT “necessity belief” draft items (29 items in total), some with similar meanings (see Table 1 and Table 2, respectively). Draft concern items encompassed NRT safety and harmfulness for the baby and self, potential side effects, perceived difficulty of access and remembering to take NRT, perceived unpleasantness to use, social embarrassment to use, getting too much or insufficient nicotine, dangers of concurrent smoking with NRT, and potential addictiveness. Draft necessity belief items encompassed perceived need for NRT for their own and baby’s health, for short-term smoking avoidance (to cope with cravings, withdrawal symptoms, stress, and trigger situations) and for longer-term smoking abstinence, and perceived need to use NRT regularly and consistently for the recommended duration. We received feedback on draft item wording from two of the three invited Patient and Public Involvement representatives; this was positive, with only one of the 29 items rephrased as a result.

**Table 1.** Baseline Item Distributions and Pre- and Post-Intervention Differences: Draft NRT Concern Items

Draft NRT concern items	Baseline (*N* = 39)	Pre- and post-intervention (*N* = 24)				Resulting item removals and amendments
	Mean(SD)	Mean(SD) pre	Mean(SD) post	Mean(SE) change	*P* ^a^	
c1. I would feel embarrassed to be seen using NRT in pregnancy	1.9(0.92)	1.5(0.66)	1.2(0.41)	−0.25(0.14)	.084	Item removed: baseline agreement <2; confirmed by intervention practitioners as not a concern
c2. I am worried that I might get more nicotine from NRT than from smoking	2.1(0.91)	2.1(1.02)	1.4(0.58)	−0.71(0.22)	.006	Amendment: “I’m worried I might get more nicotine from NRT than from smoking”
c3. Nicotine is harmful to my baby	4.3(0.92)	4.1(1.03)	2.1(1.10)	−2.04(0.29)	<.001	Item unchanged
c4. I could easily get a free supply of NRT for as long as I need it^b^ (N=37)	3.1(1.02)	3.1(1.10)	3.8(0.96)	0.75(0.28)	.013	Item removed: “don’t know” from two participants; high proportion of “3” responses; beyond intervention scope
c5. I would find it hard to remember to take NRT regularly	2.2(0.81)	2.1(0.90)	2.1(1.12)	0.00(0.19)	.963	Item removed: no sensitivity to change; reconsidered to measure self-efficacy
c6. Nicotine is the most harmful part of cigarettes	3.1(1.28)	2.9(1.38)	1.8(0.78)	−1.13(0.33)	.005	Item unchanged
c7. I am worried that NRT won’t give me enough nicotine to cope with my cravings	3.2(0.95)	3.3(1.00)	2.0(1.00)	−1.33(0.20)	<.001	Amendment: “I’m worried NRT won’t give me enough nicotine to cope with my cravings”
c8. I am worried that there will be side effects from taking NRT	3.1(0.92)	3.1(0.99)	2.2(1.09)	−0.96(0.32)	.010	Amendment: “I’m worried there will be side effects from using NRT”
c9. NRT would be unpleasant for me to use e.g., taste bad	3.1(0.81)	3.0(0.75)	2.1(1.03)	−0.83(0.26)	.007	Amendment: “NRT would be unpleasant to use e.g., taste bad”
c10. NRT is not addictive^b^	2.9(0.79)	3.0(0.88)	3.8(1.22)	0.75(0.26)	.008	Amendment, to reduce cognitive load: “not” removed: “I’m worried NRT could be addictive”
c11. NRT is safe for my baby^b^	3.4(0.74)	3.4(0.71)	4.4(0.88)	1.00(0.21)	.001	Item unchanged^b^
c12. I would want to use the least amount of NRT possible in pregnancy	3.4(0.74)	3.4(0.72)	2.5(0.98)	−0.96(0.21)	.001	Amendment, to clarify meaning: “I’d want to use only a small amount of NRT while pregnant”
c13. It is dangerous to smoke any cigarettes at the same time as using NRT	3.6(0.84)	3.7(0.86)	2.7(1.04)	−1.00(0.16)	<.001	Amendment, to align more closely with women’s concerns and behavior: “It’s dangerous to keep using NRT if I smoke during a quit attempt”
c14. I am worried that I could “overdose” on nicotine when using NRT	2.5(1.00)	2.5(1.06)	1.4(0.58)	−1.17(0.22)	<.001	Amendment: I’m worried I could “overdose” on nicotine when using NRT
c15. NRT will not be as satisfying as cigarettes	3.4(0.84)	3.3(1.01)	2.8(1.10)	−0.54(0.23)	.036	Item removed: reconsidered as likely true; beyond intervention scope

Item c10 was adapted directly from a Beliefs about Medicines Questionnaire (BMQ) concern (“I sometimes worry about becoming too dependent on my medicines”); items c2, c3, c6, c8, c11 loosely reflect a BMQ concern (“I sometimes worry about the long-term effects of my medicines”); items c1, c4, c5, c7, c9, c12, c13–15 are novel. NRT = nicotine replacement therapy.

^a^From paired Wilcoxon tests (2-tailed, *α* = 0.05).

^b^Reverse-scored item, where agreement indicates lack of concern about using NRT. A score of 5 is transformed to 1, a score of 4 to 2, and so on.

**Table 2.** Baseline Item Distributions and Pre- and Post-Intervention Differences: Draft NRT Necessity Belief Items

Draft NRT necessity belief items	Baseline (*N* = 39)	Pre-post intervention (*N* = 24)				Resulting item removals and amendments
	Mean(SD)	Mean(SD) pre	Mean(SD) post	Mean(SE) change	*p* ^a^	
n1. Using NRT instead of smoking would improve my baby’s health	4.5(0.72)	4.5(0.72)	4.7(0.70)	0.13(0.13)	.317	Amendment: baseline agreement >4. Stronger wording: “My baby’s health would improve if I used NRT”
n2. Quitting smoking would be impossible for me without NRT	4.0(0.90)	4.0(0.83)	4.2(0.82)	0.17(0.21)	.392	Item unchanged: similar wording to a validated BMQ item
n3. NRT would help me to avoid smoking in places and situations where I would usually smoke	3.8(0.81)	3.9(0.88)	4.3(0.76)	0.42(0.13)	.008	Amendment, to avoid ambiguity: “NRT will help me avoid smoking in places and situations where I’d usually smoke”
n4. NRT would relieve the discomfort (withdrawal symptoms) of quitting smoking	4.0(0.58)	4.2(0.56)	4.1(0.93)	−0.08(0.17)	.589	Amendment, to avoid ambiguity: “NRT will relieve my discomfort from quitting smoking (withdrawal symptoms)”
n5. Using NRT instead of smoking would improve my health	4.4(0.64)	4.5(0.66)	4.7(0.70)	0.17(0.16)	.305	Amendment: baseline agreement >4. Stronger wording: “My health would improve if I used NRT”
n6. For NRT to work, I’d only need to take it when I feel like I need it^b^	3.1(0.84)	3.1(0.93)	1.9(0.88)	−1.17(0.22)	<.001	Amendment: “For NRT to work, I’d only need to use it when I feel like I need it”^b^
n7. If my quit attempt is going well early on, I would want to test whether I could do without my NRT^b^	3.1(1.06)	3.1(1.10)	2.7(1.05)	−0.42(0.25)	.095	Amendment, to simplify: “If my quit attempt is going well, I’d want to test if I could do without NRT”^b^
n8. NRT would help me to avoid smoking when I’m stressed	3.6(0.85)	3.5(0.88)	3.8(0.94)	0.21(0.19)	.268	Amendment, to avoid ambiguity: “NRT will help me avoid smoking when I’m stressed”
n9. NRT would control my cravings to smoke	3.6(0.64)	3.6(0.72)	4.3(0.68)	0.67(0.16)	.001	Amendment, to avoid ambiguity: “NRT will control my cravings to smoke”
n10. NRT will not improve my chances of quitting smoking during pregnancy^b^	2.6(0.99)	2.4(1.06)	1.8(1.06)	−0.58(0.33)	.089	Amendment, to reduce cognitive load; “not” removed: “NRT will improve my chances of quitting smoking in pregnancy”
n11. NRT only works if it is taken regularly (*N* = 38)	3.7(0.67)	3.8(0.74)	4.5(0.51)	0.79(0.16)	<.001	Item removed: “don’t know” from one participant; high proportion of “3” responses; similar meaning to item “n6”
n12. NRT will help me to quit	4.2(0.47)	4.3(0.46)	4.5(0.66)	0.21(0.15)	.166	Item removed: baseline agreement >4; similar meaning to item “n10”
n13. I would no longer need NRT after a few weeks of using it^b^	2.7(0.68)	2.7(0.76)	1.8(0.64)	−0.83(0.18)	.001	Amendment, to simplify: “I’d only need to use NRT for a few weeks”^b^
n14. NRT should be taken for at least 8 weeks (*N* = 38)	3.4(0.60)	3.5(0.59)	3.9(0.93)	0.38(0.19)	.067	Item removed: “don’t know” from one participant; high proportion of “3” responses; similar meaning to item “n13”

Item n2 was adapted directly from a Beliefs about Medicines Questionnaire (BMQ) necessity belief (“My life would be impossible without my medicines”); items n1 and n5, and items n3, n4, n8–10, and n12 loosely reflect BMQ necessity beliefs (respectively, “My health, at present, depends on my medicines” and also “My health in the future will depend on my medicines” and “My life would be impossible without my medicines”); items n6, n7, n11, n13, and n14 are novel. NRT = nicotine replacement therapy.

^a^From paired Wilcoxon tests (2-tailed, *α* = 0.05).

^b^Reverse-scored item, where agreement indicates lack of perceived need for NRT. A score of 5 is transformed to 1, a score of 4 to 2, and so on.

### Phase 2: Pilot Testing of Draft Items With Pregnant Women Who Smoke

Of 39 study participants who completed the questionnaire at baseline, 24 (62%) also completed it post-intervention. At baseline, 36 participants (92%) completed all 29 pilot items; post-intervention, 24 (100%) completed all items. Table 1 and Table 2 show, respectively, the 15 draft concern items (numbered “c1” to “c15”) and the 14 draft necessity belief items (numbered “n1” to “n14”), displaying baseline distributions and pre- to post-intervention score differences (sensitivity to change). Two items were each missing one baseline response; one item was missing two baseline responses. Comments were left only for these items and only by participants who omitted them. Post-intervention changes were in the expected direction for most items, that is, in favor of NRT use, with sensitivity to change reaching statistical significance for 13 of the 15 draft concern items and 5 of the 14 draft necessity beliefs. Baseline scores were relatively high (pro-NRT) for the draft necessity belief items compared to the concern items, which appeared more normally distributed, limiting their potential to show positive post-intervention change.

Table 1 and Table 2 also show which items were removed or revised after piloting, and why. In total, seven items were removed, some in favor of better-performing items with similar meanings. Two items were removed as a result of very low (<2) or very high (>4) mean baseline agreement and no sensitivity to change (items “c1” and “n12”). Three items were removed because of appearing to assess knowledge rather than beliefs (items “c4,” “n11,” and “n14”). Two concern items were reconsidered by the research team to assess self-efficacy (item “c5”) and to be likely true (item “c15”), respectively, and were removed. Two necessities items with high baseline agreement, but of high importance based on previous research, were substantially amended to try to reduce agreement (items “n1” and “n5”). All amendments following piloting, including minor revisions intended to shorten or simplify wording and remove ambiguity, are shown in Tables 1 and 2.

### Phase 3: DCV Task

We invited 29 potential participants, of whom *N* = 16 (55%) completed the DCV task between April 29, 2021 and August 13, 2021. Other than name and email address, participant characteristics were not collected; however, all were known to the research team as postdoctoral academics with considerable expertise in smoking cessation research. Mean time for task completion was 27 minutes. Table 3 shows average confidence ratings and one-sample Wilcoxon test results per item, per construct, across all judges. Items are ordered by descending magnitude of their test statistic within each scale, that is, in descending order of DCV. Confidence ratings for 19 out of 22 items (86%) were significantly greater than 0 for their intended construct only, indicating good discriminant validity. In addition to the two non-scale (dummy) items that we believed, a priori, to measure other theoretical constructs than NRT concerns or necessity beliefs (“I’d struggle to remember to use NRT regularly”; “Doctors and midwives approve of using NRT in pregnancy”), confidence ratings for three out of 22 scale items retained from Phase 2 (14%) were not significantly greater than 0 for either construct, indicating poor DCV (“I’d want to use only a small amount of NRT while pregnant”; “My baby’s health would improve if I used NRT,” “My health would improve if I used NRT”). These three items were removed as a result, along with one item that was classed as a concern but received a low confidence rating for the alternative construct, indicating uncertainty (“I’m worried NRT won’t give me enough nicotine to cope with my cravings”). Confidence ratings were generally higher for the concern items. Most questionnaire items received no suggestions for alternative constructs, although “NRT efficacy belief” was proposed by one to two judges for some necessities items.

**Table 3.** Discriminant Content Validation (DCV) Task: Average Confidence Ratings Across Judges (*N* = 16)

Items in descending order of DCV per scale	Confidence rating: “NRT Concern”			Confidence rating: “NRT Necessity Belief”		
	Median (IQR)	*z* ^a^	*p* ^a^	Median (IQR)	*z* ^a^	*p* ^a^
**Concern items (11 items):**						
I’m worried there will be side effects from using NRT	100(0)	3.75	<.001	−95(19)	−3.52	<.001
I’m worried I could “overdose” on nicotine when using NRT	100(8)	3.70	<.001	−99(20)	−3.30	.001
I’m worried NRT could be addictive	100(8)	3.62	<.001	−91(33)	−3.54	<.001
It’s dangerous to keep using NRT if I smoke during a quit attempt	100(10)	3.62	<.001	−86(33)	−2.72	.006
I’m worried I might get more nicotine from NRT than from smoking	100(9)	3.59	<.001	−80(53)	−2.59	.010
NRT would be unpleasant to use e.g. taste bad	95(23)	3.57	<.001	−94(24)	−3.54	<.001
Nicotine is harmful to my baby	100(16)	3.46	.001	−90(27)	−2.40	.017
Nicotine is the most harmful part of cigarettes	95(36)	2.88	.004	−91(30)	−3.28	.001
NRT is safe for my baby	100(87)	2.15	.032	−90(55)	−2.79	.005
* I’m worried NRT won’t give me enough nicotine to cope with my cravings*	81(46)	2.08	.037	39(153)	−0.39	.698
* I’d want to use only a small amount of NRT while pregnant*	75(104)	1.24	.214	−39(134)	0.18	.856
**Necessity belief items (11 items):**						
Quitting smoking would be impossible for me without NRT	−93(27)	−2.81	.005	100(18)	3.54	<.001
NRT will improve my chances of quitting smoking in pregnancy	−86(49)	−2.58	.010	91(17)	3.54	<.001
NRT will help me avoid smoking in places and situations where I’d usually smoke	−90(30)	−2.87	.004	85(41)	3.53	<.001
NRT will help me avoid smoking when I’m stressed	−80(126)	−1.97	.049	80(39)	3.32	.001
NRT will control my cravings to smoke	−83(119)	−2.32	.021	83(44)	3.11	.002
I’d only need to use NRT for a few weeks	−92(128)	−2.89	.004	100(9)	3.01	.003
For NRT to work, I’d only need to use it when I feel like I need it	−86(56)	−2.85	.004	93(25)	2.91	.004
NRT will relieve my discomfort from quitting smoking (withdrawal symptoms)	−67(99)	−1.92	.055	85(50)	2.55	.011
If my quit attempt is going well, I’d want to test if I could do without NRT	−85(50)	−2.60	.009	85(36)	1.99	.046
* My baby’s health would improve if I used NRT*	15(188)	−0.10	.917	53(140)	1.19	.233
* My health would improve if I used NRT*	71(176)	−0.03	.979	75(177)	0.91	.362
**Other (dummy) items (2 items):**						
I’d struggle to remember to use NRT regularly (self-efficacy)	−10(193)	−0.11	.916	−55(159)	−1.27	.204
Doctors and midwives approve of using NRT in pregnancy (subjective norm)	56(156)	0.67	.501	−63(168)	−0.91	.364

Medians presented as distributions significantly non-normal for all confidence ratings (Shapiro–Wilk tests).

italic text denotes item removed as a result of DCV task. NRT = nicotine replacement therapy.

^a^From one-sample Wilcoxon tests (2-tailed, *α* = 0.05), where hypothesized median = 0.

## Discussion

### Main Findings

Through the processes described in this manuscript (qualitative research, item piloting, and DCV task), we derived an 18-item questionnaire intended to measure concerns and necessity beliefs about using NRT in pregnancy. Items removed following piloting and content validation included concerns about social embarrassment to use NRT, perceived ability to access NRT and to remember to use it, and necessity beliefs relating to the specifics of NRT use (for example, recommended duration), and the benefit of using NRT for own and baby’s health. These either exhibited highly pro-NRT scores at baseline among pregnant women who smoked, or they appeared to assess a different construct to ours. The 18 retained items have been classified as having good discriminant content validity, and contamination between the two constructs appears low.

The beliefs identified as important determinants of NRT adherence in pregnancy were generally well covered by the two constructs underlying the Necessities and Concerns Framework, lending support to this as a useful theory of medication adherence. Some DCV task judges commented that NRT efficacy beliefs may be a separate construct from NRT necessity beliefs, although the framework views them as part of this. Previous studies have verified the psychometric properties of the BMQ, which is based on this same framework.^14–16,32^ However, the Necessities and Concerns Framework and BMQ are concerned only with *intentional* factors underlying medication adherence, and are restricted to concerns and necessity beliefs as these are considered key. Unintentional factors fall outside of this framework, such as forgetting to use NRT or not having NRT to hand when needed, and other intentional determinants, such as self-efficacy and subjective norms, are excluded, although these form part of our broader intervention objectives.

BMQ items were difficult to adapt directly to NRT use in pregnancy, so many of our drafted items were novel or based only loosely on a BMQ item. Medications the BMQ has previously been adapted to are typically for chronic health conditions, such as asthma, hypertension, diabetes, and breast cancer.^16,17,32^ NRT has a less direct relationship with health outcomes than these, as it treats a health behavior (smoking) rather than a health condition itself, so necessity beliefs were more difficult to adapt than concerns. This would likely be the case if the BMQ was adapted to other health behaviors such as exercise or healthy eating. Most BMQ necessities items were felt to be too extreme (for example, “My health in the future will depend on my X medication,” “Without my X medication I would be very ill”). We, therefore, phrased items in terms of necessity for smoking cessation rather than for health, or for “improving” own and baby’s health, although the latter items were not retained. BMQ concern items about dependence and long-term effects were easier to adapt, overlapping with beliefs expressed by participants in our qualitative research. The larger change in draft NRT concerns than necessity belief items among our prototype NRT adherence intervention participants might suggest that concerns are more malleable, but is likely a reflection of the relatively high (pro-NRT) necessity beliefs seen at baseline among our participants, who volunteered to try NRT as part of a supported quit attempt (ie, a ceiling effect).

### Strengths and Limitations

In Phase 1 of our research, we built a strong evidence base on which to construct our questionnaire content, involving pregnant women with widely varying experiences of NRT plus other smoking cessation professionals. Previous literature was also systematically reviewed. In Phase 2, items were piloted on their target user group, that is, those undergoing a smoking cessation attempt in pregnancy with NRT plus counseling. In Phase 3 of our research, we followed good practice guidance for undertaking DCV and met recommended recruitment targets. We were also able to recruit judges with considerable construct expertise.

A potential limitation was the relatively small sample size for carrying out item analyses in our pilot study (*N* = 39 at baseline; *N* = 24 at both time points); however, it has been recommended^31^ that content validity is established before conducting studies on large numbers. Larger amounts of item-level data will be analyzed following the Smoking, Nicotine and Pregnancy 2 (SNAP2) trial in which the current questionnaire version is being used.^18^ While most items showed significant post-intervention changes in favor of NRT use, in line with key messages provided in intervention content, attrition bias may have influenced the post-intervention scores. For example, those who benefitted less from the intervention or had a less positive experience of using NRT may have been less likely to complete follow-up. It is also possible that demand characteristics might have affected post-intervention scores, though we tried to mitigate against this by separating data collection from intervention delivery as much as possible, for example, by having questionnaires completed in private and then sealed in an envelope if not carried out remotely. In terms of sample representativeness, study participation required more time and commitment than usual specialist stop-smoking support. It is therefore possible that our pilot sample had higher motivation or stronger pro-NRT beliefs at the outset of cessation support than the typical pregnant support user for whom this scale could have clinical utility. However, like typical support users, participants were under no obligation to accept NRT and were recruited in a similar way to the current opt-out system offered in England for pregnancy smoking cessation. From our recruitment rates and demographic information,^28^ we believe our sample to be typical of research participants in pregnancy cessation studies.

In our DCV task, we utilized judges with expertise in smoking cessation research. It could be argued that another important group of “expert judges” is the target population of respondents^31,33^; it may therefore be useful to confirm the content validity of items on this group, for example, using “think-aloud” methodology.^33^ Other forms of validity, such as criterion-related, are also important and are an ongoing part of our research. It is possible that our DCV task results would be less favorable had we added a further “other” construct for judges to rate; this approach has sometimes been used.^31^ However, we piloted this approach initially on the wider research team, who found the “other” construct very difficult to judge, and, after consulting with the DCV technique authors, simplified the task to our two constructs plus an optional free text box to suggest alternatives. In previous research, judges appear to avoid the “other” construct where offered,^31^ so this might be best omitted. We also added two dummy items relating to self-efficacy and social norms as comparators, which judges correctly classified as “neither construct,” supporting the validity of the technique.

### Interpretation

We believe this is the first questionnaire for measuring beliefs about NRT in pregnancy. The WI-BASC,^19,20^ while not explicitly based on the BMQ, assesses cessation medication beliefs among general smokers, and covers efficacy beliefs, stopping too soon or using too little, no point in continuing if smoking, addiction, danger to health, and difficulty using, that is, similar issues to those we discovered about NRT use during pregnancy. However, items assessing perceived need for medication exceed concerns in WI-BASC, and continuation of NRT during smoking lapses emerged as a safety concern in pregnancy rather than a lack of perceived need as in WI-BASC. Pregnant women have additional concerns about using NRT, notably their baby’s health. Our revised pregnancy NRT beliefs questionnaire has 18 items (nine per construct), scored as two separate subscales in which low concerns and high necessity beliefs indicate more pro-NRT beliefs. Further items may be removed, or a short form created, depending on the results of the SNAP2 trial and future validation work. A potential future use for the NiP-NCQ is clinical assessment of NRT beliefs at the outset of pregnancy smoking cessation treatment (for example, within specialist pregnancy stop smoking support), and tailoring support to address negative beliefs. Study practitioners and pregnant participants in our intervention optimization work have found the questionnaire helpful for exploring and discussing NRT-specific beliefs during the initial stop-smoking consultation.

## Conclusions

We developed an 18-item self-report questionnaire that measures concerns and necessity beliefs in pregnancy about using NRT. These are beliefs that can be targeted to try to improve treatment adherence and, potentially, pregnant women’s chances of quitting smoking. Retained items have good discriminant content validity and initial sensitivity to change appears promising. Further validation work is ongoing and will explore whether scores predict adherence behavior.

## Supplementary Material

A Contributorship Form detailing each author’s specific involvement with this content, as well as any supplementary data, are available online at https://academic.oup.com/ntr.

ntad030_suppl_Supplementary_Material

## Data Availability

Anonymised data for Phases 2 and 3 of this research are available upon reasonable request from the corresponding author.
