# Supplementary material for: Development and Content Validation of a Questionnaire for Measuring Beliefs About Using Nicotine Replacement Therapy for Smoking Cessation in Pregnancy
Source: Nicotine Tob Res. 2023 Mar 2;25(7):1310–8. doi: 10.1093/ntr/ntad030 (PMC10256886; doi:10.1093/ntr/ntad030)
Supplement: ntad030_suppl_Supplementary_Material [file ntad030_suppl_supplementary_material.docx]

**Definitions of constructs presented to Discriminant Construct Validation task participants:**

***Concerns*** about medications are defined as “concerns about a range of potential adverse consequences” (i.e. beliefs that reflect anxiety or worry about using NRT to quit smoking in pregnancy).    ·

***Necessity beliefs*** about medications are defined as “perceptions of personal need for treatment” (i.e. beliefs that NRT is personally necessary/needed for quitting smoking in pregnancy).
